# Cross-Cultural Analysis of Volition: Action Orientation Is Associated With Less Anxious Motive Enactment and Greater Well-Being in Germany, New Zealand, and Bangladesh

**DOI:** 10.3389/fpsyg.2018.01043

**Published:** 2018-06-28

**Authors:** Monischa B. Chatterjee, Nicola Baumann, Danny Osborne, Shamsul H. Mahmud, Sander L. Koole

**Affiliations:** ^1^Differential Psychology, Personality Psychology and Psychological, University of Trier, Trier, Germany; ^2^School of Psychology, University of Auckland, Auckland, New Zealand; ^3^Department of Psychology, University of Dhaka, Dhaka, Bangladesh; ^4^Faculty of Behavioural and Movement Sciences, Clinical Psychology, and Mental Health, VU University Amsterdam, Amsterdam, Netherlands

**Keywords:** action orientation, volition, motive enactment, well-being, cross-cultural psychology

## Abstract

**Background:** People differ in action vs. state orientation, that is, in the capacity for volitional action control. Prior research has shown that people who are action-rather than state-oriented are better able to perceive and satisfy own motives (e.g., affiliation, achievement, power), which translates into greater psychological well-being (Baumann et al., 2005; Baumann and Quirin, 2006). However, most of the extant literature has been limited to samples from European countries or the US. To address this shortcoming, the present paper investigated the associations between action vs. state orientation, psychological well-being, and anxious style of motive enactment among samples in Germany, New Zealand, and Bangladesh (combined *N* = 862).

**Methods:** To examine the consistency of our results across countries, a multi-group structural equation model (SEM) was used to examine the associations between action orientation, anxious motive enactment, and well-being. Subsequent mediation analyses assessed whether anxious motive enactment mediated the relationship between action orientation and well-being across each of the three samples.

**Results:** Across all three cultural groups, action orientation was associated with less anxious motive enactment and higher well-being. Moreover, mediation analyses revealed significant indirect paths from action orientation through less anxious motive enactment to well-being that were similar across the three samples.

**Conclusions:** These findings suggest that individual differences in action vs. state orientation have a similar psychological meaning across Western and non-Western cultures.

## Introduction

The capacity for volitional action control is present within all human beings. However, not everyone is equally proficient at this capacity (Kuhl and Beckmann, 1994). So-called “action-oriented” people display high levels of efficiency at volitional action control. When the going gets tough, action-oriented people are able to self-regulate their own emotions intuitively (e.g., downregulation of negative and/or upregulation of positive affect), maintain access to their own motives (e.g., affiliation, achievement, and power), and enact motives in a flexible and efficient manner. Thus, action orientation is a volitional mode that is highly conducive to well-being. In contrast, “state-oriented” people display pronounced volitional deficits particularly when challenged by demanding or stressful situations. Under stress, state-oriented people become trapped in negative emotional states and enact motives rather anxiously.

To date, the major part of research on action vs. state orientation has been conducted on Western cultures. Consequently, little is known about the generalizability of the correlates of action vs. state orientation to non-Western cultures. In the present article, we aim to address this oversight. In what follows, we begin by briefly reviewing the current literature on action vs. state orientation, which is based on research within Western cultures. Next, we introduce a cross-cultural perspective on action vs. state orientation. Finally, we present the findings of a cross-cultural empirical study that examined the correlates between action vs. state orientation with motive enactment and well-being in Germany, New Zealand, and Bangladesh.

## Research on action vs. state orientation

The notion of action vs. state orientation derives from German volitional psychology (Kuhl, 1981, 2000, 2001; see also Baumann et al., 2018). Action orientation is defined as a meta-static mode of control that facilitates the enactment of change-oriented intentions (Kuhl, 1984). In contrast, state orientation is defined as a cata-static mode of control in which the status quo is preserved by inhibiting the enactment of change-oriented intentions (Kuhl, 1984).

Since the notion of action vs. state orientation was introduced more than three decades ago, individual differences in action vs. state orientation have become the focus of hundreds of published studies (for reviews, see Kuhl and Beckmann, 1994; Diefendorff et al., 2000; Koole et al., 2012). Action-oriented people, relative to state-oriented people, have been found to be better able to self-regulate affect (Brunstein, 2001; Baumann and Kuhl, 2002; Koole and Jostmann, 2004), maintain well-being, life satisfaction, body vitality, and health, especially under adverse conditions (Baumann et al., 2005; Herrmann and Brandstätter, 2013; Wojdylo et al., 2014b; Schlinkert and Koole, 2017a). Moreover, compared to their state-oriented counterparts, action-oriented people display better psychological functioning in a wide variety of domains including work performance (Diefendorff, 2004; Wojdylo et al., 2014a), athletic prowess (Heckhausen and Strang, 1988; Beckmann and Kellmann, 2004), educational achievement (Jaramillo and Spector, 2004; Schlüter et al., 2017), economic behavior (Bagozzi et al., 1992), health behavior (Palfai, 2002; Schlinkert and Koole, 2017b), and relationship satisfaction (Backes et al., 2017).

One consistent pattern of results across different domains is that action-oriented people, compared to state-oriented people, are better able to perceive and enact their own motives for affiliation (e.g., striving to establish and maintain warm and friendly interpersonal relations), achievement (e.g., striving to meet a standard of excellence), and power (e.g., striving to impact and influence others). Action-oriented people tend to commit themselves to goals that are more congruent with implicit motives (Brunstein, 2001; Baumann et al., 2005) and enact motives more often in intrinsic and integrative ways than do state-oriented people (Hofer and Busch, 2011; Baumann et al., 2016). Moreover, action-oriented people enact their goals less often in controlled and anxious ways than do state-oriented people (Baumann and Quirin, 2006; Schlinkert and Koole, 2017b; Wolf et al., 2018).

The aforementioned patterns of goal commitment and goal enactment that characterize action-oriented people appear to be highly conducive to well-being (Brunstein et al., 1998; Deci and Ryan, 2000; Hofer and Busch, 2013). For instance, Baumann et al. (2005) found that congruence between implicit and explicit motives partially mediates the effect of action orientation on well-being and recovery from psychosomatic complaints. Likewise, Baumann and Quirin (2006) found that a less anxious enactment of explicit motives partially mediates the relationship between action orientation and health—a central finding and blueprint for our current study.

The aforementioned self-report measure of anxious motive enactment taps into (a) automatic perceptual orientation toward incongruent and undesirable events, (b) anticipation of negative outcomes, and (c) behavioral inhibition. Thus, anxious motive enactment signifies a focus on problems and the frustration of personal needs. According to Kuhl (2000, 2001), such a problem focus originates from negative affect. Action vs. state orientation, in contrast, captures individual differences in the ability to self-regulate (attenuate) and leave negative affective states once they are aroused. Because action-oriented individuals do not use their self-regulatory ability unless the task or context requires it, they also experience negative affect and display anxious motive enactment at times.

Research on action vs. state orientation to date has mostly been conducted among people who were born and raised in Western countries. Indeed, only a handful of studies have investigated the effects of action vs. state orientation in non-Western countries such as China (Bagozzi et al., 1992; Song et al., 2006) and Ecuador (Jaramillo et al., 2007). Furthermore, we located only two cross-cultural studies in this area. In both studies, similar associations between action orientation and indicators of achievement motivation and academic performance were observed among German and Vietnamese students (Helmke and Tuyet, 1999) and among Croatian, German, and Japanese students (Niemivirta et al., 2001). As far as we know, no cross-cultural studies have examined the effects of action vs. state orientation on motive enactment and well-being. This neglect of cultural context, which remains common in contemporary psychology (Henrich et al., 2010; Aldao, 2013), begs the question whether individual differences in action vs. state orientation have the same psychological meaning in non-Western cultures.

## Cultural psychology perspectives

### Independence-interdependence and autonomy

To derive hypotheses for the present research, we turned to broad research traditions within (cross-)cultural psychology that are theoretically relevant to action vs. state orientation. To these ends, one research tradition within cultural psychology has emphasized cultural differences in independent vs. dependent self-concepts (Markus and Kitayama, 1991) or the related notion of individualism-collectivism (Triandis, 1989). This tradition is relevant because action-oriented people tend to have a more independent self-concept than do state-oriented people (Olvermann et al., 2004). Yet, the constructs of independence (i.e., conceptions of the self as distinct and separate; Markus and Kitayama, 1991) and autonomy (i.e., striving because one identifies with it; Deci and Ryan, 2000) differ from each other (Chirkov et al., 2003) and are not the same as action orientation (i.e., regulating emotions and actions through the self). Nevertheless, all three relate to the self.

In an influential article, Markus and Kitayama (1991, p. 226) wrote, “In many Western cultures, there is a faith in the inherent separateness of distinct persons. […] Achieving the cultural goal of independence requires construing oneself as an individual whose behavior is organized and made meaningful *primarily by reference to one's own internal repertoire of thoughts, feelings, and action* rather than by reference to the thoughts, feelings, and actions of others. The essential aspect of this view involves a conception of the self as an autonomous, independent person […]. We assume that, on average, relatively more individuals in Western cultures will hold this view than will individuals in non-Western cultures.” This argument suggests that independence is valued less in the East than in the West. According to this perspective, action orientation would be a hallmark of Western cultures.

Despite its popularity, empirical research does not support the notion of systematic East-West differences in independence. Oyserman et al. (2002; see also Fiske, 2002) undertook a comprehensive review and meta-analysis of cultural-comparative research on self-concept, well-being, attribution style, and relationality. This review revealed highly heterogeneous findings, such that different scales produced different results and large differences between presumably homogeneous countries (e.g., Japan and Korea; Fiske, 2002). Cultural differences between East and West thus appear to be “neither as large nor as systematic as often perceived” (Oyserman et al., 2002, p. 40). However, Oyserman and colleagues' review focused on mean level differences in the content of people's self-conceptions. Thus, it remains possible that the functional meaning of process variables like action orientation differs between Western and non-Western cultures. For example, if action orientation is less valued in Eastern cultures, it may not offer the same kinds of advantages as in Western cultures. Similarly, it is conceivable that Eastern cultures compensate for the volitional deficits of state-oriented people (e.g., through social support).

A second research tradition associated with cross-cultural psychology has emphasized the notion of autonomy, defined as the capacity for free and volitional self-regulation (Deci and Ryan, 2000, 2017). This literature is relevant because action-oriented people tend to be higher in autonomous self-regulation than are state-oriented people (e.g., Koole and Jostmann, 2004; Baumann and Scheffer, 2011; see also Koole et al., 2018).

Based on self-determination theory (SDT; Deci and Ryan, 2000, 2017), researchers within this tradition have suggested that the functional meaning of autonomy is similar across Western and non-Western cultures. Consistent with this view, several cross-cultural studies have shown that autonomous goal striving is equally important for well-being across cultures (e.g., Chirkov et al., 2003; Sheldon et al., 2004; Church et al., 2013). For example, among over 1,700 people from Belgium, China, the USA, and Peru, satisfaction of autonomy needs predicted well-being, whereas frustration of autonomy needs predicted psychological problems (Chen et al., 2015). Cross-cultural similarity is also evident in other processes (e.g., actor effect; Krettenauer and Jia, 2013).

In addition to studies drawing on SDT, the cross-cultural importance of autonomy is further supported by research on motive congruence. When people autonomously choose their goals, their goals should be congruent with their implicit needs (Baumann et al., 2005). To these ends, several studies have shown that the pursuit of motive-congruent goals is associated with greater well-being across both Western and non-Western cultures (for a review, see Hofer and Busch, 2013). Because motive congruence and autonomous goal striving are characteristic of action- rather than state-oriented individuals, a disposition toward action orientation should have similar consequences across different cultural contexts.

## The present research

We designed the present study to empirically examine the association between action orientation and self-regulatory outcomes in Western and Eastern cultures. Comparing Western and Eastern cultures was of particular interest, given that some cultural psychologists have emphasized East-West differences in constructs related to independence/interdependence (e.g., Markus and Kitayama, 1991) constructs that coincide with action vs. state orientation. However, as discussed above, more recent evidence suggests that cultural differences between the East and West may be less pronounced with regard to independence/interdependence (Oyserman et al., 2002). Consequently, we expected to find mostly cross-cultural continuity in the present study, consistent with findings on cross-cultural continuity in the value of autonomous self-regulation (e.g., Church et al., 2013; Hofer and Busch, 2013).

Specifically, the present study examined university undergraduates from Germany, New Zealand, and Bangladesh. To our knowledge, no study to date has investigated the effects of action orientation in New Zealand and Bangladesh. In the selection of the three nationalities, we followed the recommendations by Berry et al. (1997) to look for patterns among cultures with large differences and, at the same time, also investigate cultural differences in countries with cultural similarities. Specifically, Germany and New Zealand are both Western cultures that share many cultural values and have a common linguistic background (i.e., English and German are both Germanic languages). Yet, the countries differ in dimensions relevant for cross-cultural psychology (e.g., individualism-collectivism and power distance dimensions; for an overview see Hofstede and Hofstede, 2006)^1^

In all three cultural samples, we measured individual differences in action orientation, anxious motive enactment, and subjective well-being. We predicted that action orientation would be associated with greater well-being (*H*1) and less anxious motive enactment (*H*2). Furthermore, we predicted that variations in anxious motive enactment would mediate the relationship between action orientation and well-being (*H*3). Finally, we predicted that the link from action orientation through less anxious motive enactment to greater well-being would generalize across our participants from Germany, New Zealand, and Bangladesh (*H*4).

## Methods

### Participants and procedure

Data were collected at the University of Trier (Germany), the University of Auckland (New Zealand), and the University of Dhaka (Bangladesh). The samples consisted of undergraduate students of psychology. Altogether, 862 participants completed the questionnaires measuring action vs. state orientation, anxious motive enactment, and well-being in their native language: German, English, and Bengali, respectively. Afterwards, participants answered questions regarding demographic information. As a precondition to take part in the survey, students had to be raised in the country in which they were attending university: 282 students (206 female, 73%) raised in Germany^2^, 332 students (256 female, 77.1%) in New Zealand, and 248 (118 female, 47.6%) in Bangladesh. In total, participants' age ranged from 17 to 54 (*M* = 22.15; *SD* = 4.04). The majority of students came from middle-class families (82.60%). Taken together, the different cultural samples shared a similar socioeconomic and educational background (e.g., middle-class families). In return for their participation, participants could either receive course credit, a small monetary compensation, or entry into a prize draw for a $100.00 voucher.

### Materials

The German sample took part in a German version of the study and the sample from New Zealand in an English version of the study. All questionnaires had been used in several studies before in the respective languages. For the survey material used with the Bangladesh sample, two independent researchers translated all materials from English into Bengali and back into English until they reached full agreement over the translations.

#### Action vs. state orientation

We used the Action Control Scale (ACS-24; Kuhl, 1994) to measure individual differences in action vs. state orientation. The validity of the ACS-24 has been supported by over 100 published studies (for comprehensive reviews, see Kuhl and Beckmann, 1994; Diefendorff et al., 2000; Koole et al., 2012). The ACS-24 has two main subscales, each consisting of 12 items. The failure—(or threat)—related subscale measures action vs. state orientation in coping with threatening situations, and the decision—(or demand)—related subscale measures action vs. state orientation in coping with demanding situations. In the present study, our theoretical focus was on action vs. state orientation in general. Moreover, in preliminary statistical analyses, we found that failure- and decision-related action vs. state orientation yielded very similar results. Therefore, we combined the two subscales into one scale that measures general action vs. state orientation.

For each item, participants are presented an affectively charged event (e.g., failure, demand). Therefore, the scale does not assess how often/easily individuals *enter* affective states (affect sensitivity), but rather, whether they are able to *leave* such states (affect regulation) and terminate rumination and hesitation (Baumann et al., 2007). Two illustrative items that measure action orientation are: “When several things go wrong on the same day (a) I just keep on going as though nothing had happened, or (b) I usually don't know how to deal with it.” And “When I know I must finish something soon (a) I find it easy to get it done and over with, or (b) I have to push myself to get started.” For each of the 24 items, participants were asked to select the option that applies to their typical reaction. In both examples, option “a” represents an action-oriented response, whereas option “b” represents a state-oriented response. The number of action-oriented responses was summed so that the total score could range from 0 to 24, with lower scores indicating lower action orientation (i.e., state orientation) and higher scores indicating higher action orientation. The internal consistencies (Cronbach's alpha) of the scale were α = 0.82 (Germany), α = 0.80 (New Zealand), and α = 0.77 (Bangladesh).

#### Anxious motive enactment

We used the Motive Enactment Test (MET; Kuhl and Henseler, 2004) to measure anxious enactment of affiliation (e.g., “I feel paralyzed when faced with rejection”), achievement (e.g., “No matter how good my performance is, I still see critical aspects”), and power motives (e.g., “I often feel inadequate around authoritative people”) with four items each. Participants rated how much these statements applied to them on a 4-point scale (1 = “*not at all*”; 4 = “*in full*”). In contrast to the ACS items, MET items relate to specific motive themes and do not systematically start with a negative or frustrating event. Anxious motive enactment was calculated as the sum of the 12 items. As such, the total score could range from 12 to 48. Although the items refer to different content domains (i.e., affiliation, achievement, and power), the global anxious motive enactment index had high levels of internal consistency in the three samples: α = 0.87 (Germany), α = 0.83 (New Zealand), and α = 0.68 (Bangladesh).

#### Well-being

The WHO-Five Well-Being Index (WHO, 1998) was used to measure subjective well-being. The index consists of five items (*During the last 2 weeks: “I have felt cheerful and in good spirits”; “I have felt calm and relaxed”; “I have felt active and vigorous”*; “*I woke up feeling fresh and rested”*; “*my daily life has been filled with things that interest me”*). Participants were asked to rate their well-being over the last 2 weeks on a 6-point scale (0 = “*at no time*”; 5 = “*all of the time*”). The items were summed to form a single measure of well-being that could range from 0 to 25. In the present sample, the internal consistencies of this measure were α = 0.81 (Germany), α = 0.86 (New Zealand), and α = 0.79 (Bangladesh).

## Results

### Descriptive information

To test for significant differences in the mean levels of our main study variables between the three cultural samples, we conducted a multivariate analysis of variance (MANOVA) with culture as an independent variable and action orientation, anxious motive enactment, well-being, and age as dependent variables. As listed in Table 1, the New Zealand sample scored significantly higher in anxious motive enactment and was, on average, younger than the other two samples. Additionally, compared to the German, but not the Bangladeshi, sample, the New Zealand sample scored lower in well-being.

**Table 1:** Descriptive Statistics of Main Study Variables for the Three Sample Groups.

	**Germany**	**New Zealand**	**Bangladesh**		
	***M*** **(*****SD*****)**	***M*** **(*****SD*****)**	***M*** **(*****SD*****)**	***F***_(2, 859)_	η2
Action orientation	11.44^a^ (5.15)	11.69^a^ (4.87)	11.42^a^ (4.60)	0.29	0.00
Anxious motive enactment	25.53^a^ (6.75)	29.14^b^ (6.35)	26.49^a^ (5.28)	28.17^***^	0.06
Well-being	14.13^a^ (4.63)	12.96^b^ (5.02)	13.15^ab^ (5.16)	4.78^**^	0.01
Age	23.12^a^ (3.85)	20.96^b^ (5.04)	22.65^a^ (1.68)	25.73^***^	0.06

*The action orientation scale ranges from 0 to 24, anxious motive enactment from 12 to 48, and well-being from 0 to 25*.

a, b *Means in the same row that do not share subscripts differ at p < 0.05 in post hoc (Scheffe) tests*.

^*^p < 0.05,

** *p < 0.01*,

*** *p < 0.001*.

The correlations between the main study variables for the total sample and within the samples of each country are listed in Table 2. Consistent with *H*1 and *H*2, action orientation correlated positively with well-being, but negatively with anxious motive enactment, across all three samples. Furthermore, in all samples, less anxious motive enactment was significantly associated with greater well-being.

**Table 2:** Correlations of the measured variables within the total sample (upper half: above the diagonal), the German sample (upper half: below the diagonal), the New Zealand sample (lower half: above the diagonal), and the Bangladeshi sample (lower half: below the diagonal).

	**AO**	**AME**	**WB**	**Age**	**Gender^a^**
Action orientation (AO)		−0.51^***^	0.36^***^	0.05^**^	0.16^***^
Anxious motive enactment (AME)	−0.53^***^		−0.33^***^	−0.10^**^	−0.16^***^
Well-being (WB)	0.38^***^	−0.36^***^		−0.02	0.03
Age	0.08	−0.08	−0.07		0.08^*^
Gender^a^	0.23^***^	−0.29^***^	0.11	0.16^**^	
Action orientation (AO)		−0.61^***^	0.41^***^	0.06	0.07
Anxious motive enactment (AME)	−0.40^***^		−0.34^***^	−0.03	−0.09
Well-being (WB)	0.29^***^	−0.23^***^		−0.04	−0.01
Age	0.04	0.03	−0.02		0.00
Gender^a^	0.24^***^	−0.03	0.02	0.08	

a *female = 1; male = 2*.

* *p < 0.05*,

** *p < 0.01*,

*** *p < 0.001*.

### Cross-cultural measurement equivalence

In order to identify possible group-dependent sources of non-invariance and to test whether the concepts measured have the same meaning in the national samples included in our study, we conducted confirmatory factor analyses (CFA) for each instrument in each country and, as a multigroup confirmatory factor analysis (MGCFA), across all countries (Lee et al., 2011; Meuleman and Billiet, 2011). In the MGCFA, when the measurement models across groups have the same factor structure, the scales are configurally invariant (Steenkamp and Baumgartner, 1998; Lee et al., 2011; Meuleman and Billiet, 2011). Additionally, metric (factor loading) invariance is obtained if the factor loadings of the items on the underlying construct they are supposed to measure are invariant across countries. Notably, metric invariance is sufficient for comparisons that are based on difference scores, such as regression coefficients or correlational relationships across cultures. Finally, scalar invariance (i.e., similar item intercepts across samples) is preferred when scale means will be compared (Steenkamp and Baumgartner, 1998; Fischer and Fontaine, 2011; Meuleman and Billiet, 2011).

Because the goal of the present study was to compare relations (rather than means) between the countries, demonstrating metric invariance (i.e., the equality of factor loadings) was sufficient. Table 3 displays the fit statistics for the various measurement models. As shown here, the measurement of well-being demonstrated metric invariance (i.e., ΔCFI < 0.01; see Cheung and Rensvold, 2002). For the remaining two measurement models, the change in CFI exceeded the criteria established by Cheung and Rensvold. Nevertheless, the model fit for the action orientation scale ACS-24 and the anxious motive enactment scale of the MET ranged from acceptable to very good both within national samples and for the metric (and configural) invariant measurement models (see Table 3).

**Table 3:** Model Fit Indices for Confirmatory Factor Analyses (CFA) in the Three Sample Groups and the Multigroup Confirmatory Factor Analysis (MGCFA) across countries.

	**Action orientation**		**Anxious motive enactment**		**Well-being**	
	**RMSEA (90% CI)**	**CFI**	**RMSEA (90% CI)**	**CFI**	**RMSEA (90% CI)**	**CFI**
Germany	0.054 (0.047/0.062)	0.892	0.071 (0.056/0.087)	0.940	0.171 (0.128/0.218)	0.912
New Zealand	0.058 (0.051/0.065)	0.837	0.078 (0.064/0.092)	0.904	0.142 (0.102/0.185)	0.954
Bangladesh	0.036 (0.025/0.046)	0.899	0.084 (0.068/0.101)	0.731	0.172 (0.126/0.222)	0.890
**CROSS-CULTURAL**						
Configural invariance	0.051 (0.046/0.056)	0.914	0.078 (0.069/0.087)	0.899	0.169 (0.144/0.194)	0.914
Metric invariance	0.052 (0.048/0.057)	0.793	0.083 (0.075/0.091)	0.867	0.134 (0.115/0.154)	0.909

### Mediation model

Our main interest was not in mean-level differences between the cultural samples, but rather, in the underlying functional relationships between our study variables. Therefore, we standardized all variables within each cultural group and tested if anxious motive enactment mediates the relationship between action orientation and well-being. To these ends, we conducted a mediation analysis with 5,000 bootstrap resamples (without replacement) using the SPSS macro Model 4 described by Hayes (2012, 2013) and computed a point estimate and a 95% confidence interval (CI) for the mediation effect. In pursuing these analyses, we controlled for participants' age and gender.

#### Total sample

**Figure 1:**
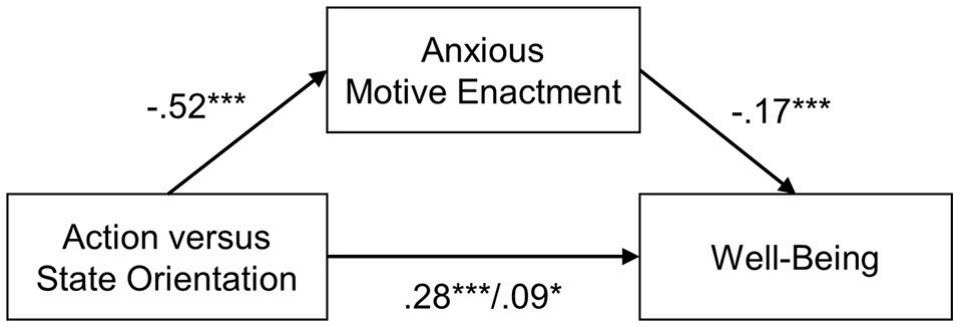
Mediation model with the direct effect of action vs. state orientation on well-being and the indirect effect through anxious motive enactment. *limits of the 95% confidence interval do not include zero ***p < 0.001.

In the mediation analysis for the total sample, the mediator variable model yielded a significant main effect of action orientation on anxious motive enactment (see Table 4). Consistent with H2, action orientation was associated with less anxious motive enactment. The model accounted for ~28% of variance in anxious motive enactment, *R*2 = 0.28, *F*_(3, 858)_ = 109.66, *p* < 0.001. In the dependent variable model (see Table 4), there were significant main effects of action orientation and anxious motive enactment on well-being. Consistent with H1, action orientation was associated with greater well-being. Furthermore, anxious motive enactment was associated with lower well-being. The model accounted for ~13% of the variance in well-being, *R*2 = 0.16, *F*_(4, 857)_ = 40.52, *p* < 0.001. The statistical significance of the indirect effect of the mediation model was verified with bootstrapped standard errors and 95% confidence intervals (CIs), as the 95% CI did not include zero. Thus, consistent with *H*3, the relationship between action orientation and well-being was (partially) mediated by less anxious motive enactment (see Figure 1).

**Table 4:** Summary of the Direct Effect of Action Orientation (Predictor) on Well-being (Outcome) and the Indirect Effect of Action Orientation through Anxious Motive Enactment (Mediator) on Well-being for the Whole Sample (Controlling for Gender and Age).

	**Mediator variable model (DV** = **Need Satisfaction)**			**Dependent variable model (DV** = **Well-Being**^*****^a^*****^**)**			**Direct effect of action orientation on well-being**^**^a^**^			**Indirect effect of action orientation on well-being**^**^a^**^			
	***B***	***SE***	***t***	***B***	***SE***	***t***	***B***	***SE***	***t***	***b***	***SE***	**Boot.LLCI**	**Boot.ULCI**
Constant	−0.14	0.18	−0.80	0.42	0.19	2.20							
Action orientation	0.52	0.03	17.54^***^	0.28	0.04	7.67^***^	0.28	0.04	7.67^***^				
Anxious motive enactment				−0.17	0.04	−4.65^***^				0.09	0.02	0.05	0.13

*LLCI (ULCI), Lower (Upper) Limit of Confidence Interval*.

a *Modified WHO-Index with four items*.

^*^p < 0.05,

^**^p < 0.01,

*** *p < 0.001*.

To examine whether a particular domain of anxious motive enactment was particularly responsible for mediating the relationship between action orientation and well-being, we conducted an additional mediation analysis in which anxious enactment of achievement, affiliation, and power motives were entered simultaneously as three independent mediators. Although action orientation was associated with less anxious motive enactment in all three domains (*F*s ≥ 36.44, *p*s < 0.001), only one of the specific indirect effects was significant. Specifically, whereas the specific indirect effect of action orientation on well-being through less anxious enactment of power motives was significant, the remaining two specific indirect effects through less anxious enactment of achievement and affiliation motives were non-significant.

#### Separate cultural samples

Additional mediation analyses tested whether similar relationships would emerge in each of the three samples. Consistent with *H*4, the global index of anxious motive enactment mediated the link between action orientation and well-being in all three countries. Thus, there was cross-cultural convergence in the functional meaning of action orientation. In Germany, New Zealand, and Bangladesh, action orientation was associated with less anxious motive enactment and, in turn, greater well-being.

When entering anxious enactment of affiliation, achievement, and power motives simultaneously as three independent mediators, action orientation was associated with less anxious motive enactment in all motive domains and across samples (*F*s > 3.05, *ps* < 0.05). Furthermore, the total indirect effect from action orientation on well-being through less anxious motive enactment was significant across samples. There were, however, some cultural variations in which particular motive(s) mediated the link between action orientation and well-being. In the German sample, less anxious enactment of power and achievement motives mediated the relationship between action orientation and well-being, but not less anxious enactment of affiliation motives (i.e., the 95% CI for the indirect effect of action orientation on well-being included zero). In the New Zealand sample, only less anxious enactment of power motives mediated the relationship between action orientation and well-being. In the Bangladeshi sample, less anxious motive enactment in one domain alone did not mediate the relationship between action orientation and well-being. Taken together, not all three motive domains mediated the link between action orientation and well-being for all cultural samples. However, these observed cultural variations did not correspond to a simple East-West distinction. Accordingly, it is likely that at least some of these cultural variations were due to chance. Overall, the results support the mediating role of motive enactment strategies in the relationship between action orientation and well-being in different motive domains in all three samples.

### Structural equality

Finally, we tested the equivalent structures of the relationships between action orientation, anxious motive enactment, and well-being, first in each sample, and then in a multi-group structural equation model (SEM). In the SEM analyses, the measurement models for action orientation, anxious motive enactment, and well-being were the same as those used in the cross-cultural measurement equivalence analyses. To examine the consistency of our findings across countries, we specified two models: The first model was an unconstrained model in which no equality constraints were imposed on the data across samples. This model also allowed the exogenous variables to correlate with each other. The second model constrained the paths between our focal variables to be equal in all three samples. Structural equivalence of the mediation model across our three groups would be supported if the addition of these equality constraints to the unconstrained model do not produce a significant decrease in model fit.

The model fit of the unconstrained model was χ2 = 4,523.58, *df* = 2,332; *p* < 0.001; RMSEA = 0.057 (90% CI = 0.055/0.060); CFI = 0.729; SRMR = 0.075. According to Hu and Bentler (1999), SRMR values close to (or below) 0.08, RMSEA values close to or below 0.06 and CFI values close to (or higher than) 0.90 indicate acceptable model fit. In our data, only the CFI departed from these standard criteria for acceptable model fit. However, scholars note that the fit indices should be interpreted holistically. Moreover, the aim of these analyses was to see if our mediational model differed across countries. The constrained model differed significantly from the unconstrained model (χ2 = 4,679.66, *df* = 2,411; *p* < 0.001; RMSEA = 0.057 (90% CI = 0.055/0.060); CFI = 0.719; SRMR = 0.080; Δ χ2 = 156.08, Δ*df* = 79; *p* < 0.001). Yet, given that at least two items of one latent construct (i.e., the item that is fixed at a unity to identify the model and one other item) are equivalent, cross-national comparisons can be made (Byrne et al., 1989; Steenkamp and Baumgartner, 1998). In other words, partial equivalence requires invariance of some, but not all, factor loadings. Modification indices (that provide information about model changes when some parameters are not held equal across sample groups) showed that freely estimating some factor loadings (one item of the ACS-24 in the New Zealand sample and three items of the anxious motive enactment scale of the MET)—rather than constraining them to equality across sample groups—substantially improved the model. Accordingly, the χ^2^ difference test indicated that the structural weights of the constrained model did not differ significantly from the unconstrained model with partial equivalence Δ χ2 = 86.61, Δ*df* = 68; *ns* (χ2 = 4,604.37, *df* = 2,396; *p* < 0.001; RMSEA = 0.057 (90% CI = 0.054/0.059); CFI = 0.727; SRMR = 0.078; Δχ2 = 80.79, Δ*df* = 79; *p* < 0.001). Again, the fit-indices showed a satisfactory match of the structural weights model with the data. As before, only the CFI departed from these standard criteria for acceptable model fit. Taken together, the findings show that the model was largely consistent across all three cultural samples.

## Discussion

In the present research, we examined the association between action vs. state orientation, anxious motive enactment, and well-being among people in Germany, New Zealand, and Bangladesh. Across all three cross-cultural samples, action orientation was positively associated with less anxious motive enactment and greater well-being. Moreover, the relationship between action orientation and well-being was mediated by less anxious enactment of motives (affiliation, achievement, and power) across all three cultures. Taken together, these findings provide the strongest and most systematic evidence to date for the cross-cultural generalizability of the effects of action vs. state orientation on well-being.

The present findings inform recent debates about cultural variations in the valuation of independence. According to an influential perspective on cross-cultural research, independence is a value that is widely celebrated in the West, but not in the East (Markus and Kitayama, 1991). A meta-analytic review of cross-cultural psychological research, however, found no evidence of a straightforward East-West distinction in people's self-conceptions (Oyserman et al., 2002). The present findings are consistent with the latter meta-analytic findings given that our sample from Bangladesh did not describe themselves in less action-oriented terms than did our samples from Germany and New Zealand. Of course, action orientation is not the same as independence. Whereas, independence relates to the *content* of self-conceptions, action orientation focuses on self-regulatory *processes*. Therefore, in line with the process nature of action orientation, we were especially interested in the functional meaning of action orientation across cultures rather than in mean level differences between cultures.

In the present research, action orientation correlated with anxious motive enactment and well-being similarly across our one Eastern and two Western samples. Irrespective of participants' national origins, our action-oriented participants enacted social motives less anxiously and, in turn, had greater well-being compared to our state-oriented participants. These findings are consistent with prior studies in non-Western countries showing equivalent effects of action orientation as in Western countries (Bagozzi et al., 1992; Song et al., 2006; Jaramillo et al., 2007) and two cross-cultural studies showing similar effects of action orientation on achievement motivation and academic performance across cultures (Helmke and Tuyet, 1999; Niemivirta et al., 2001). The available evidence thus points to the following conclusion: the meaning of action vs. state orientation is quite similar across different cultures.

In addition to identifying important cross-cultural similarities, we found some potential differences between cultures. When analyzing the mediating role of anxious motive enactment separately for the three motive themes, results varied across cultures. In Germany, anxious enactment of power and achievement motives were significant mediators between action orientation and well-being. In contrast, only anxious enactment of power mediated this association within the New Zealand sample, and no single motive domain uniquely mediated the association between action orientation and well-being in Bangladesh. One possible explanation for these cultural differences is that shorter subscales are less reliable. Alternatively, power motives and their non-anxious enactment may be especially important in Western cultures in which hierarchies are (at least perceived to be) more permeable. In Bangladesh, in contrast, hierarchies are perceived to be rather steep and fixed in Bangladesh (Hofstede and Hofstede, 2006). Therefore, specific power motives may not be more important for well-being than other motives.

In the present study, we found no evidence that state-oriented people in an Eastern culture are better able to overcome their volitional problems, at least no more so than state-oriented people in Western cultures. This is not to say, however, that state-oriented people are beyond redemption. Prior research has shown that adverse effects of state orientation are mitigated when positive close relationships are made salient (Koole et al., 2005; Puterman et al., 2010; Koole and Fockenberg, 2011; Chatterjee et al., 2013, 2017). Indeed, when conditions are sufficiently supportive, state-oriented people may even outperform action-oriented people (Koole et al., 2005) and experience more positive emotions (Van Putten, 2015). Thus, state-oriented people are not necessarily destined to poor life outcomes as long as adequate support is available.

## Limitations and future perspectives

Because the present study is the first cross-cultural investigation of the effects of action orientation on motive enactment and well-being, there are some limitations that need to be resolved in future research. First, the present study is exclusively based on correlational, cross-sectional data. Hence, the causal directions of the observed relationships remain uncertain. Although action vs. state orientation represents a stable disposition and findings from a longitudinal field study show that state orientation predicts increases in goal-related conflict over time (Wolf et al., 2018), it is conceivable that (at least in some cases) repeated or permanent frustration of social motives may induce (rather than follow from) state orientation. Longitudinal research is needed to resolve the causal relationships between individual differences in volition, motive enactment strategies, and well-being.

Second, prior work indicates that action- and state-oriented individuals do not differ in their ability to access their motives and enact them adequately under relaxed and friendly conditions (Baumann and Kuhl, 2003; Koole and Jostmann, 2004; Baumann et al., 2005; Jostmann and Koole, 2006; Chatterjee et al., 2013, 2017). Consequently, state orientation is only maladaptive in the face of stress. Momentary stress could act as an intermediary mechanism that moderates anxious motive enactments and its focus on events that frustrate (rather than satisfy) needs. In future studies, it would be informative to experimentally induce stress and explore the nature of different stress levels on volitional action control and well-being across countries.

Finally, in the present investigation, we included only samples with similar socioeconomic and educational backgrounds (e.g., being students and coming from middle-class families). Whereas the present samples might have held more independent orientations, ethnic minorities and lower income groups are likely to have stronger interdependent orientations (Greenfield et al., 2003; Kagitcibasi, 2005; Hofstede and Hofstede, 2006). This fact complicates the attempt to capture the complete spectrum of reality of lives. Thus, it remains important to test whether the present findings extend to non-student samples as well as other cultural groups and nationalities. In addition, it will be important for future research to examine potential differences between motive domains.

## Conclusion

The present study is the largest and most systematic cross-cultural analysis to date on individual differences in action vs. state orientation. Our findings indicate that there is a considerable degree of cross-cultural convergence on the psychological significance of action vs. state orientation for both motive enactment and well-being. Nevertheless, more work is required before psychologists can definitively conclude that the functional significance of action vs. state orientation is similar across cultures. We hope that the current study provides the foundations for such future endeavors.

Although no one can say what the future will hold, we can make some educated guesses about the likely outcomes of cross-cultural research on action vs. state orientation. First, it seems unlikely that future research will show that action orientation is psychologically less beneficial in the East than in the West. The East-West distinction does not appear to correspond with any meaningful psychological dimension (Fiske, 2002). Indeed, as shown in the present study, the relationship between action vs. state orientation and well-being through less anxious motive enactment was equivalent across cultures. Therefore, the time seems ripe to move beyond the simple East-West dichotomy characterizing much of modern cross-cultural research (Vignoles et al., 2016).

We also anticipate that future research will uncover more subtle ways in which culture influences action vs. state orientation. Theoretically, a disposition toward action orientation emerges from repeated socialization experiences (especially early in life) in which responsiveness to affective self-expression is encouraged (e.g., when parents respond promptly and adequately with soothing in the case of sadness/anxiety, or encouragement in the case of frustration). Thereby, externally supported emotion regulation styles gradually turn into the ability to self-regulate affect (Kuhl, 2000; Kuhl et al., 2015). Cultural variations in socialization can thus be expected to shape dispositions toward action vs. state orientation in many ways (even though these ways do not appear to differ between the East and West). Uncovering the cultural dimensions that can explain variance in action vs. state orientation is an important challenge for future research.

In sum, our findings indicate that the functional meaning of action vs. state orientation is similar across Western and Eastern cultures. In our samples containing participants from both the East and the West, action- compared to state-oriented individuals enacted personal motives less anxiously which, in turn, translated into greater well-being. Volitional action control thus appears to be a human capacity that supports beneficial self-regulatory outcomes for Easterners and Westerners alike. Examining how people conduct their lives in different cultural contexts may thus help to uncover universal principles of human nature (Baumeister, 2005).

## Ethics statement

This study was conducted in accordance with the ethics regulations of the Vrije Universiteit Amsterdam, the German Society for Psychology (DGPs), and comparable ethical standards. The protocol was approved by the Scientific and Ethical Review Board of the Vrije Universiteit Amsterdam [VCWE-2015-150 (RENEWAL)]. All participants gave informed consent in accordance with the Declaration of Helsinki.

## Author contributions

MC collected the data in Germany, was involved in conducting the statistical analyses and writing the first draft of the manuscript. NB was involved in conducting the statistical analyses and writing the draft of the manuscript. DO was involved in collecting the data in New Zealand and contributed with supportive input regarding the statistical analyses. SM collected the data in Bangladesh. SK completed the final draft of the manuscript.

### Conflict of interest statement

The authors declare that the research was conducted in the absence of any commercial or financial relationships that could be construed as a potential conflict of interest.

## Footnotes

^1^The three countries differ in psychological aspects such as Hofstede's classical dimensions of individualism and power distance (both ranging from 0 to 100). In individualism, Bangladesh (20) scores lower than Germany (67) and New Zealand (79). In power distance (i.e., the degree to which hierarchy and power differences are tolerated and considered normal in society), Bangladesh (80) scores considerably higher than Germany (35) and New Zealand (22). Although Hofstede‘s cultural dimensions have been criticized (e.g., Schwartz, 1994; Sinha and Tripathi, 1994; Oyserman et al., 2002), the indices provide a glimpse of the cultural breadth of our sample.

^2^Data from 152 of the 282 German participants were included in an additional study. After having completed the online survey, these participants were invited to take part in an additional experiment that was conducted during the same time period. Results from this experiment (i.e., recovery from a negative mood induction) are reported in Chatterjee et al. (2013; Study 2).
